# Supplementary material for: Physician perceived barriers and facilitators for self-measured blood pressure monitoring- a qualitative study
Source: PLoS One. 2021 Aug 20;16(8):e0255578. doi: 10.1371/journal.pone.0255578 (PMC8378703; doi:10.1371/journal.pone.0255578)
Supplement: S1 File — (DOCX) [file pone.0255578.s001.docx]

**Supplementary data 1- Identifying Barriers and Facilitators to Home BP monitoring Using the Theoretical Domains Framework**

**Interview Guide**

*Prioritize questions marked with asterisk if time pressures limit the number of questions asked

**Introduction:**

Thank you for agreeing to participate in this interview. I am working with Dr. Gupta on the QI on HTN management study. The aim of this QI is to help us understand better ways of managing HTN. With that goal, we are trying to find out how we can facilitate use of home BP monitoring in our health system. Your responses will help us understand how best to implement home BP monitoring in practice.

All of your responses will remain confidential and will only be reported in aggregate. The interview will be recorded and transcribed for analysis.

Do you have any questions before we begin?

**Practice (current hypertension management):**

1. How do you manage hypertension?

*Prompts:*

At what BP do you start or change BP medications?

How do you follow BPs after the medication changes?

When do you follow up patients after altering medications?

What is your BP goal?

1. What difficulties do you encounter when managing hypertension?

*Prompts:*

Time, pushback from staff;

Compensation

Patient adherence- medication side effects, lack of knowledge

1. *Do you use home BP monitoring? (*intentions*)

-If no, what prevents you from using home BP monitoring?

*Prompts:*

Do not feel the need. No evidence. Not practical. No reimbursement. No time. No resources.

**Nature of the Behavior:**

1. *(if you use home BP monitoring) I’d like you to walk me through the steps you take in using home BP monitoring. (describe the process)

*Prompts:*

How? do you provide patients with the paper-based blood pressure sheet. if yes, what do you do with the data that you get back? How often?

How do you treat?

Which patients?

How do you follow home BP readings?

BP goals?

**The innovation (home BP monitoring with remote management with or without clinical pharmacists):**

1. *What do you think about home BP monitoring? (*Beliefs about consequences*)

*Prompts:* (*Beliefs about consequences*)

How will home BP monitoring change patient outcomes? benefits to patients

Your practice? -following guidelines, achieve better BP

1. *Do you have any reservations about home BP monitoring?

*Prompts:*

ineffective,

patients do not know how to check BP,

no way to follow up BP in between clinic visits,

lack of protocols for managing home BP

1. Are there any difficulties with home BP monitoring?

*Prompts:* cost, time taken away from other tasks

1. Do you that feel the benefits of using home BP monitoring outweigh the costs?

How so?

1. mychart BP tool for patients:
2. Have you ever ordered mychart home BP readings for patients? (yes no)

*Prompts:*

-If no, why not?

*Prompts:* i didn't know it existed, i don't know how to order it, i don't know what to do with the results, i started using it but the results became overwhelming, i order it, but don't know where to find the results (smart phrase, episodes), other.

-If yes, have you found it useful? How?

*Prompts:* control of BP, white coat hypertension (elevated when coming to clinic), other

1. Do you have workflow support in place to assist you in managing the patient-entered results?

-If no, do you think you would use it more if there was more support (for monitoring the returned results and creation of follow up plans)

-If yes, do you find it helpful?

*Prompts*- saves time

1. Do you see any other barriers to using this tool?

**Motivation:**

1. *What might motivate you to increase the use of home BP monitoring (if already using)? (if not already using home BP monitoring, ….start using home BP monitoring)

*Prompts:*

reminders to follow home BP.

having a protocol in place.

Feedback on achieved BP?

Physician Incentives? (e.g. compensation)

1. *In ideal circumstances, to what extent would you like to use home BP monitoring in your practice in the future? (Goals)
2. How likely is that to happen? (optimism)

*Prompt:*

If no, how can we make it happen?

1. *What do experts in the HTN field say about using home BP monitoring? (Social influences)

*Prompts:* e.g., senior colleagues, clinical leaders, guidelines

1. Do these opinions influence your decision of whether or how you use home BP monitoring?

*Prompts:*

If you got the sense that others didn’t approve of home BP monitoring, would that influence whether or how you use it?

1. How do clinical practice guidelines support or detract from your use of home BP monitoring?

*Prompts:*

build your belief in the treatment?

How do you feel about its quality, clarity, supporting evidence, consistency with other guidelines? (of guidelines on home BP monitoring),

1. What emotions do you experience when using home BP monitoring? (Emotions)

*Prompts:*

Positive/negative emotions?

In your opinion, how would someone new at using home BP monitoring feel? E.g., hopeful, optimistic, resentful, stressed, anxious, content, excited?

**Opportunity**

1. *How often do you see patients who you think you would like to recommend home BP monitoring for?
2. Do you think home BP monitoring is underutilized?
3. *In your opinion, should home BP monitoring be done by a PCP or someone like a nurse practitioner, nurse, or pharmacist helping the PCP?
4. Would some other health care provider (eg: NP, PA, pharmacist) be better suited for home BP monitoring?
5. What if we trained pharmacist to monitor home BP and manage them with a health system approved protocol? i.e. a pharmacist receiving home BP readings and modifying therapy according to a hospital approved protocol.
6. Will addition of a pharmacist to follow home BP and assist you with management of BP **in between clinic appointments** be helpful? How?
7. We could even transmit home BP to the pharmacists electronically? The pharmacists will be trained to manage HTN on their own but could contact you in difficult cases.
8. * What are the main barriers and facilitators for you using home BP monitoring with assistance from a clinical pharmacist?
9. *What would you recommend for overcoming these barriers or formalize these facilitators?

*Prompts:*

automated consults/ electronic prompts vs regular consult to pharmacy

**Capability:**

1. What are some of the skills needed by someone to be able to help with home BP monitoring?

*Prompts:*

What skills would someone who is chosen to manage home BP monitoring need to have or develop?

1. Do you think a pharmacist has these skills?

If no- can they be trained to do it?

1. Will you use home BP monitoring more often if you had help from a trained pharmacist?
2. Can you think of other strategies to improve HTN management? (relative advantage)

Prompts:

what is the advantage of any competing approaches?

**Conclusion:**

1. *That’s all the questions I have for you, has anything occurred to you about this topic that I haven’t asked about?
