# Supplementary material for: Physician perceived barriers and facilitators for self-measured blood pressure monitoring- a qualitative study
Source: PLoS One. 2021 Aug 20;16(8):e0255578. doi: 10.1371/journal.pone.0255578 (PMC8378703; doi:10.1371/journal.pone.0255578)
Supplement: Attachment — Submitted filename: Response to reviewers.docx [file pone.0255578.s004.docx]

We thank the editors and the reviewers for their thoughtful feedback. Our responses to the concerns are outlined below.

**Reviewer #2**

1) This manuscript, entitled “Physician perceived barriers and facilitators for self-measured blood pressure monitoring- A qualitative study”, is an important contribution to the literature for self-measured blood pressure monitoring. The authors have done a nice job addressing previous reviewer comments.

Response: Thank you.

2) Suggest reviewing it again for grammar editing.

Response: Thank you for pointing out errors, typos and unclear sentences. We have reviewed the entire manuscript again and corrected these errors according to your suggestions.

3) Under the Opportunity section, the authors state “Physicians saw an opportunity for SMBP. Under Social Influences, most physicians identified published evidence as the major reason for using SMBP, and not peer influence. Since SMBP is backed by evidence, some PCPs found it concerning if others did not use SMBP. Only a change in evidence, and not the behavior of their colleagues, would make them not use SMBP (Table 3, social influences).” The finding that is more interesting than what would make SMBP users change their practice is what would make SMBP non-users change their practice. The way this paragraph is written, there is no opportunity on which we can act.

Response: We agree. We have rephrased this section.

Other changes:

We added some more demographic details on primary care providers (requested in the previous review) which we were now able to obtain from our health system.

We removed the abbreviation ‘HTN’ for hypertension.
