# Supplementary material for: Physician perceived barriers and facilitators for self-measured blood pressure monitoring- a qualitative study
Source: PLoS One. 2021 Aug 20;16(8):e0255578. doi: 10.1371/journal.pone.0255578 (PMC8378703; doi:10.1371/journal.pone.0255578)
Supplement: Attachment — Submitted filename: PONE-D-20-38491 reviewer comments 6-16-21.docx [file pone.0255578.s003.docx]

This manuscript, entitled “Physician perceived barriers and facilitators for self-measured blood pressure monitoring- A qualitative study”, is an important contribution to the literature for self-measured blood pressure monitoring. The authors have done a nice job addressing previous reviewer comments.

A few minor comments:

1. Suggest reviewing it again for grammar editing – found multiple punctuation (e.g. inconsistent use of Oxford comma), spelling (e.g. “specifc”, “fulfil”), and grammar mistakes (e.g. missing hyphens in compound adjectives like “patient care related”, “evidence based”). Proper grammar and editing will add needed clarity throughout.
2. Suggest editing the sentence “A majority of patients are Caucasian, 6% Hispanic, 2.5% Asian, 1.7% Black, and 18% unknown or other.” to “The racial/ethnic breakdown of patients was XX% Caucasian, 6% Hispanic, 2.5% Asian, 1.7% Black, and 18% unknown or other.
3. “Approximately 49% of all patients with a BP reading had atleast…” – need a space in ‘at least’.
4. You use both “White” and “Caucasian” in describing race in your study. Suggest picking one (White).
5. Under the Opportunity section, the authors state “Physicians saw an opportunity for SMBP. Under Social Influences, most physicians identified published evidence as the major reason for using SMBP, and not peer influence. Since SMBP is backed by evidence, some PCPs found it concerning if others did not use SMBP. Only a change in evidence, and not the behavior of their colleagues, would make them not use SMBP (Table 3, social influences).” The finding that is more interesting than what would make SMBP users change their practice is what would make SMBP non-users change their practice. The way this paragraph is written, there is no opportunity on which we can act.
6. “Under Behavioral Regulation respondents elaborated differences in using SMBP for managing HTN.” Need the word “on” after “elaborated”.
7. “Despite their beliefs in the strong evidence behind SMBP, there was underutilization of SMBP due to logistical barriers, lack of resources and trained personnel to help with SMBP.” – need an “and” before “lack”
8. “…suggesting need for better strategies to monitor SMBP” – need the word “the” before “need”
9. “(such as use of patient portal for entering SMBP)” – need “the” or “a” before “patient portal”
10. “performance assessments, standardized protocols” – need an “and” after the comma
11. “With stakeholders; patients, physicians, health systems and insurers interested in adopting SMBP, practical strategies that are scalable are needed.” – this is an awkward sentence. Suggest rewrite of “With stakeholders such as patients, physicians, health systems, and insurers interested in adopting SMBP, practical, scalable strategies are needed.”
12. Table 2 says SMPB instead of SMBP
